# Effect of the BiZact™ Low-Temperature Dissecting Device on Intra- and Postoperative Morbidities Related to Tonsillectomy—A Systematic Review and Meta-Analysis

**DOI:** 10.3390/medicina60091415

**Published:** 2024-08-29

**Authors:** Yun Jin Kang, Gulnaz Stybayeva, Se Hwan Hwang

**Affiliations:** 1 Department of Otorhinolaryngology-Head and Neck Surgery, Soonchunhyang University College of Medicine, Cheonan 14584, Republic of Korea; savie87@gmail.com; 2 Department of Physiology and Biomedical Engineering, Mayo Clinic, Rochester, MN 55905, USA; stybayeva.gulnaz@mayo.edu; 3 Department of Otolaryngology-Head and Neck Surgery, Bucheon St. Mary’s Hospital, College of Medicine, The Catholic University of Korea, Seoul 06591, Republic of Korea

**Keywords:** tonsillectomy, pain, adverse effect, meta-analysis

## Abstract

*Background and Objectives*: We investigated the effects of using a BiZact™ device for tonsillectomy on operating time, intraoperative blood loss, postoperative bleeding rate, and pain through a meta-analysis of the relevant literature. *Materials and Methods*: We reviewed studies retrieved from the databases of PubMed, SCOPUS, Google Scholar, Embase, Web of Science, and Cochrane up to March 2024. The results were analyzed following PRISMA guidelines. Six studies that compared the outcomes of patients receiving perioperative BiZact™ tonsillectomy with those in control groups (cold steel dissection or bipolar tonsillectomy) were included for this analysis of the outcomes, which included intraoperative bleeding and time, postoperative pain, and frequency of postoperative bleeding. *Results*: The operative time (SMD −11.5985, 95%CI [−20.3326; −2.8644], I^2^ = 99.5%) in the treatment group was significantly reduced compared to the control group. However, BiZact™ showed no significant efficacy in reducing intraoperative bleeding when compared with the control group (SMD −0.0480, 95%CI [−1.8200; 1.7240], I^2^ = 98.6%). Postoperative pain on day 1 (SMD −0.0885, 95%CI [−0.4368; 0.2598], I^2^ = 98.9%), day 3 (SMD −0.2118, 95%CI [−0.6110; 0.1873], I^2^ = 99.5%), and later than day 7 (SMD 0.0924, 95%CI [−0.2491; 0.4338], I^2^ = 98.6%) in the treatment group was not significantly reduced relative to the control group. When compared to the control group, BiZact™ did not reduce the incidence of secondary postoperative bleeding control in the operation room (OR 0.5711, 95%CI [0.2476; 1.3173], I^2^ = 32.1%), primary bleeding (OR 0.4514, 95%CI [0.0568; 3.5894], I^2^ = 0.0%), or all postoperative bleeding events (OR 0.8117, 95%CI [0.5796; 1.1368], I^2^ = 26.3%). *Conclusions*: This study demonstrated that using the BiZact™ device for tonsillectomy significantly decreased the operative time but could not effectively reduce intraoperative bleeding or postoperative pain and bleeding.

## 1. Introduction

Tonsillectomy is one of the most common surgeries worldwide [1,2]. Postoperative pain is a major concern following tonsillectomy. Although it has a relatively short operative time, it may involve postoperative bleeding and pain, along with a delayed return to a normal diet [3]. Post-tonsillectomy bleeding and pain remain an important issue [4]. The optimal surgical method and surgical device have yet to be determined and remain an active area of study [5]. Post-tonsillectomy pain intensity is dependent on the type of tonsillectomy, intra- and postoperative medications, and tissue damage from bleeding control [6,7,8]. Cold steel dissection, a traditional tonsillectomy method, uses instruments to remove the palatine tonsil and ligate the blood vessels with sutures [5,9]. Hot dissection with electro-cauterization, coblation, or a diathermia scissor is also widely used [1]. Tonsillectomy using electro-cauterization has the advantage of achieving reductions in surgical time and intraoperative bleeding, but there is also a risk of post-tonsillectomy pain or bleeding [10]. About 1.3–20% of patients reported post-tonsillectomy bleeding [11].

The BIZact™ (Medtronic, Mansfield, MA, USA) is a new surgical device for vessel sealing that takes continuous measurements of tissue impedance, and which received approval in 2016 [12,13]. BiZact™ consists of a 12 cm shaft and a curved jaw that provides easy access to the tonsillar bed [12]. When the tissue is sufficiently sealed between the jaws, an acoustic signal is generated and the tissue is cut [12]. Tissue damage is minimized by delivering minimal bipolar energy to the remaining tissues around the tonsil [13]. A major feature is the reduction in thermal spread to the surrounding tissues [12]. Tonsillectomy using this device is ultimately a safe surgery with a short surgical time and relatively short learning curve [14].

There have been various studies confirming the effectiveness of BiZact™ on pediatric tonsillectomies [14,15,16,17], and its effectiveness has been proven in comparison to LigaSure™ [18]. However, it involves a higher cost than using another bipolar device for tonsillectomy, and there is no statistical certainty that patient morbidity will be reduced [15,16]. There has been no meta-analysis that comprehensively compares and analyzes these pros and cons. To our knowledge, this is the first meta-analysis comparing tonsillectomy using BiZact™ with cold steel dissection or bipolar tonsillectomy in terms of operating time, intraoperative blood loss, and postoperative bleeding rate and pain.

## 2. Materials and Methods

The Preferred Reporting Items Guidelines for Systematic Review and Meta-Analysis were followed when conducting this systematic review and meta-analysis [19]. This study protocol was prospectively registered in the Open Science Framework as follows: https://osf.io/uxv7p/ (accessed on 1 April 2024).

### 2.1. Search Strategy and Study Selection

The Population, Intervention, Comparison, Outcomes, and Study (PICOS) details were as follows: (1) Population: patients who underwent tonsillectomy. (2) Intervention: tonsillectomy using the BiZact™ device. (3) Comparison: a bipolar tonsillectomy or cold steel dissection. (4) Outcomes: operative time, intraoperative bleeding, postoperative pain grading, and incidence of postoperative bleeding. (5) Study design: prospective or retrospective study. Clinical studies published prior to March of 2024 were identified from Pubmed, SCOPUS, Google Scholar, Embase, Web of Science, and the Cochrane Register of Controlled Trials. The following key search terms were used: ‘bizact’, ‘tonsillectomy’, ‘adenotonsillectomy’, ‘vessel sealing devices’, ‘pain’, ‘operative time’, and ‘bleeding’.

**Figure 1:**
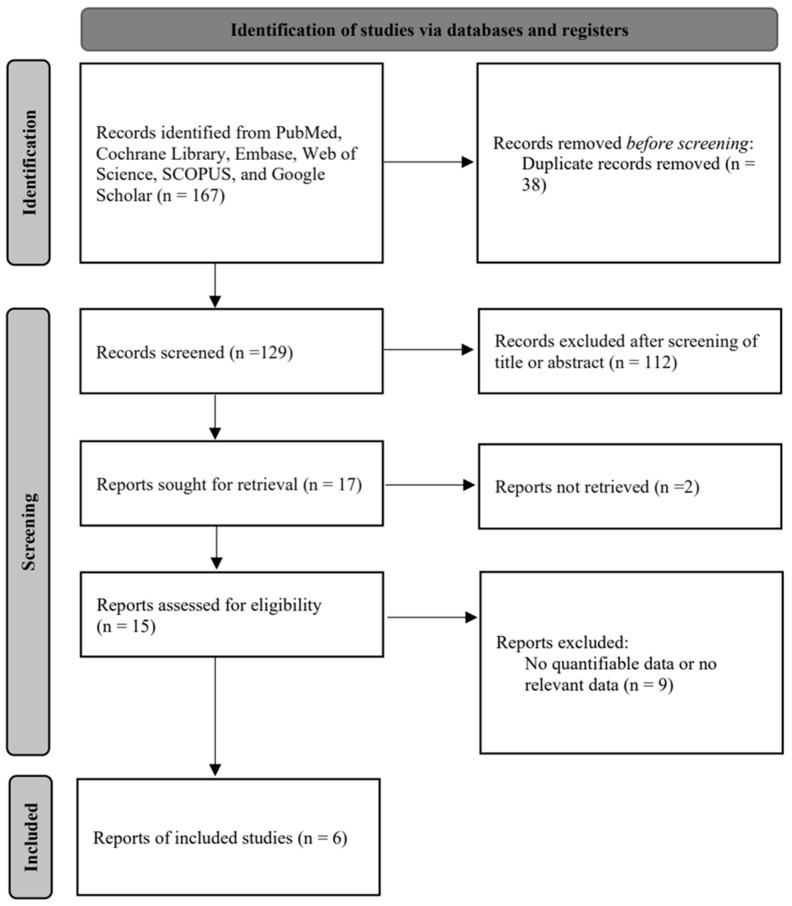
Study selection diagram.

The database search proceeded with the aid of a librarian with more than 10 years of experience, and the authors additionally searched the references listed in the retrieved articles to ensure that there were no missing reports. Two independent reviewers (YJK and SHH) screened all abstracts and titles for the candidate studies and discounted the studies not associated with tonsillectomy using a BiZact™ device. The full texts of potentially relevant studies were used if the decision regarding inclusion could not be made from the abstract alone. In the case of differing opinions, inclusion or exclusion was decided by discussion with a third reviewer (GS). Studies that satisfied the next inclusion criteria were considered to be eligible for review: trials that studied patients undergoing tonsillectomy using the BiZact™ device. We did not include patients who underwent concomitant procedures along with tonsillectomy, such as sleep surgery. Studies were also excluded from the analysis if the outcomes of interest were not clearly provided with quantifiable data, or if it was impossible to evaluate the appropriate data from the published results. Figure 1 summarizes the search strategy used to identify the studies selected for the meta-analysis [1,5,12,20,21,22].

### 2.2. Data Extraction and Risk of Bias Assessment

Data from eligible studies were extracted using standardized forms [23]. The analyzed outcomes were as follows: intraoperative bleeding (grading or amount), operative time, postoperative pain grading reported by patients, and incidence of postoperative bleeding (total bleeding event, primary bleeding (in 24 h after tonsillectomy), bleeding control in operation room). These outcomes were compared between the treatment group and the control group (patients that used no treatment or saline injection) during the perioperative period. From the studies marked for inclusion, we selected data regarding patient number, pain grading scale, operative time, amount of intraoperative bleeding, incidence of postoperative bleeding, and the *p*-value, which was reported in the form of a comparison between the treatment group and the control group. This was conducted to determine the influence of BiZact™ on intra- and postoperative morbidities.

### 2.3. Analyses for Statistics

The statistical analysis of the included studies was conducted using the R-4.3.1 program (R Software Foundation, Vienna, Austria). For quantitative variables, the meta-analysis was conducted using the mean difference (MD) or standardized mean difference (SMD). The SMD was adopted as a summary statistic to standardize the results of the studies to an equal scale when the studies measured the equal outcome but assessed it in various methods. In the incidence of postoperative bleeding, the odds ratio (OR) was calculated. Heterogeneity was calculated using the I^2^ test, which describes the rate of variation across studies that can be attributed to heterogeneity rather than probabilistic chance. The measure ranges from 0 (no heterogeneity) to 100 (maximum heterogeneity). All results were reported with 95% confidence intervals (CI), and all *p*-values were two-tailed. When significant heterogeneity among outcomes was found (defined as I^2^ > 50), the random-effects model was used as described by DerSimonian–Laird. This model assumes that the true treatment effects in individual studies may be different from one another, and that these are normally distributed.

Subgroup analysis was also performed. Those outcomes that did not present a significant level of heterogeneity (I^2^ < 50) were analyzed with the fixed-effects model. The fixed-effects model uses the inverse variance approach, and it is assumed that all studies come from a common population.

We used a funnel plot and Egger’s test concurrently to identify potential publication bias. We also used Duval and Tweedie’s trim and fill to compensate for the summed effect size with respect to publication bias. Moreover, to estimate the effect of each individual study in the overall meta-analysis results, sensitivity analyses were conducted. These analyses were performed by repeating the meta-analyses while omitting a different study each time.

## 3. Results

In total, six studies with 1880 participants were included and reviewed for this meta-analysis. The study characteristics are presented in Table 1 and the results of the bias evaluations are presented in Supplementary Tables S1 and S2.

### 3.1. Effect of BiZact™ Tonsillectomy on Intraoperative Time and Bleeding Compared with the Control Group

**Figure 2:**
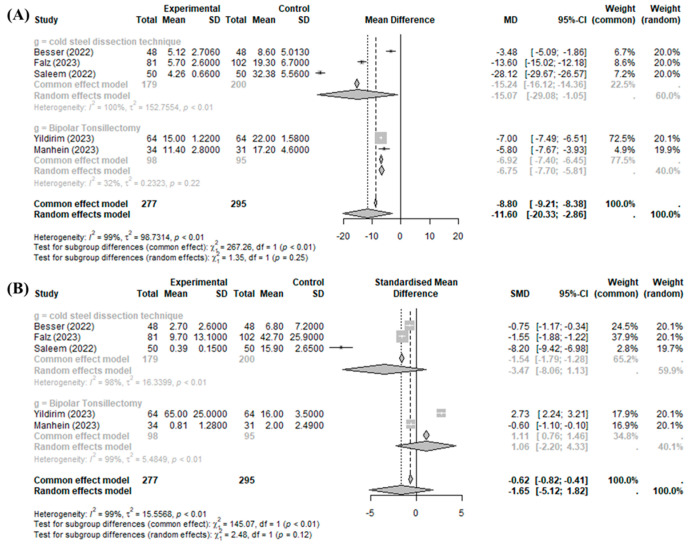
BiZact™ tonsillectomy versus conventional tonsillectomy: (**A**) mean difference in operative time and (**B**) standardized mean difference in intraoperative bleeding. SD: standardized deviation, MD: mean difference, CI: confidence index, SMD: standardized mean difference.

The BiZact™ device was demonstrated to be effective in reducing intraoperative time when compared with the control group (MD −11.5985 min, 95%CI [−20.3326; −2.8644], I^2^ = 99.5%), but it could not reduce intraoperative bleeding (SMD −0.0480, 95%CI [−1.8200; 1.7240], I^2^ = 98.6%) (Figure 2).

Significant inter-study heterogeneity (I^2^ > 50%) was found for the above outcomes. The overall analysis did not consider the particular definition of the control group (bipolar tonsillectomy or cold steel dissection). This omission is reflected in the high heterogeneity (more than 50%) of the results obtained by all studies. In the subgroup analysis of the operative time compared to those of cold steel dissection and bipolar tonsillectomy, operative time was improved with the BiZact™ device (compared to cold steel dissection: MD −15.0662, 95%CI [−29.0798; −1.0526]; compared to bipolar tonsillectomy: MD −6.7545, 95%CI [−7.7033; −5.8058]). By contrast, in the subgroup analysis of operative bleeding compared to those of cold steel dissection and bipolar tonsillectomy, bleeding risk was not improved with the BiZact™ device in any comparison (compared to cold steel dissection: SMD −3.4671, 95%CI [−8.0626; 1.1284]; compared to bipolar tonsillectomy: SMD 1.0637, 95%CI [−2.2006; 4.3281]).

### 3.2. Effect of BiZact™ Tonsillectomy on Pain Grading Compared with the Control Group

**Figure 3:**
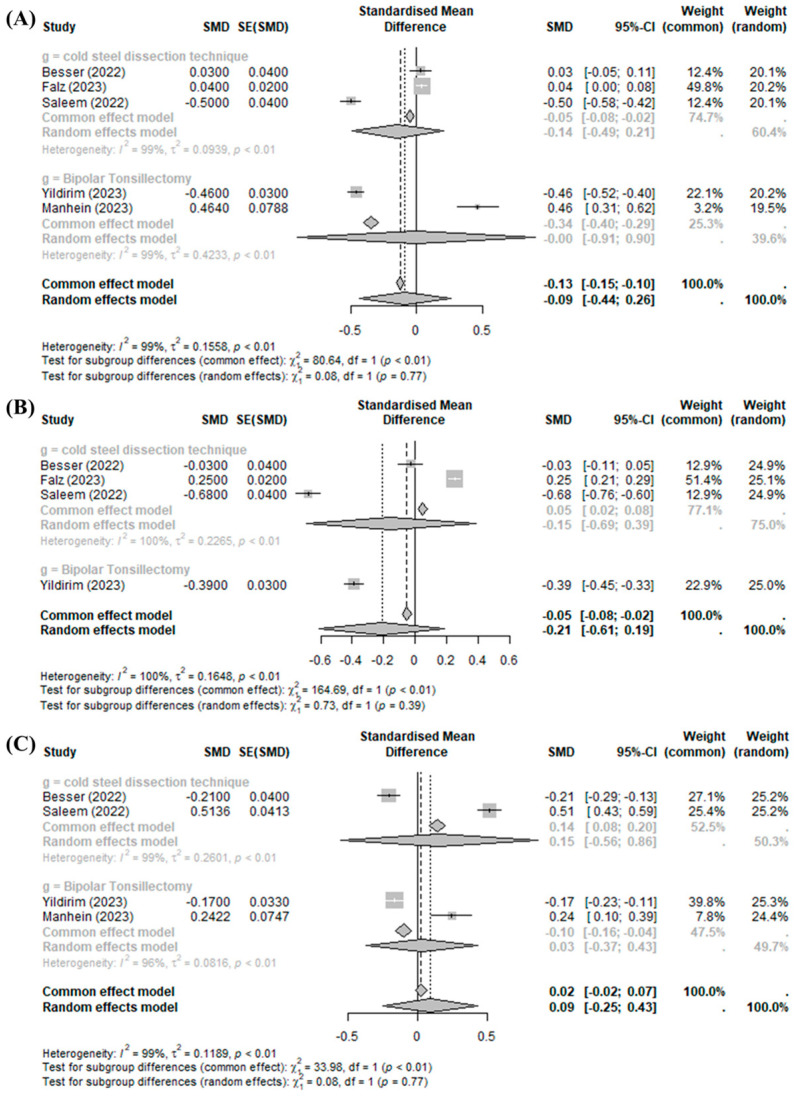
BiZact™ tonsillectomy versus conventional tonsillectomy: standard mean difference in postoperative pain score on (**A**) day 1, (**B**) day 2, and (**C**) later than day 7. SMD: standardized mean difference, SE: standard error, CI: confidence index.

Tonsillectomy using BiZact™ did not show a significant effect on postoperative pain on day 1 (SMD −0.0885, 95%CI [−0.4368; 0.2598], I^2^ = 98.9%), day 3 (SMD −0.2118, 95%CI [−0.6110; 0.1873], I^2^ = 99.5%), and later than day 7 (SMD 0.0924, 95%CI [−0.2491; 0.4338], I^2^ = 98.6%) when compared with the control group (Figure 3).

Significant inter-study heterogeneity (I^2^ > 50%) was found at the above outcomes. In the subgroup analysis of post-tonsillectomy pain compared to cold steel dissection, the pain score was not improved with the BiZact™ device in any time period of postoperative follow-up (day 1 (SMD −0.1426, 95%CI [−0.4914; 0.2063]), day 3 (SMD −0.1526, 95%CI [−0.6926; 0.3873]), and later than day 7 (SMD 0.1517, 95%CI [−0.5574; 0.8608])). However, compared to bipolar tonsillectomy, the BiZact™ device significantly reduced postoperative pain on day 3 (SMD −0.3900, 95%CI [−0.4488; −0.3312]); there were no significant differences in postoperative pain on day 1 (SMD −0.0009, 95%CI [−0.9064; 0.9046]) or later than day 7 (SMD 0.0307, 95%CI [−0.3732; 0.4345]).

### 3.3. Effect of BiZact™ Tonsillectomy on the Incidence of Postoperative Bleeding Compared with the Control Group

**Figure 4:**
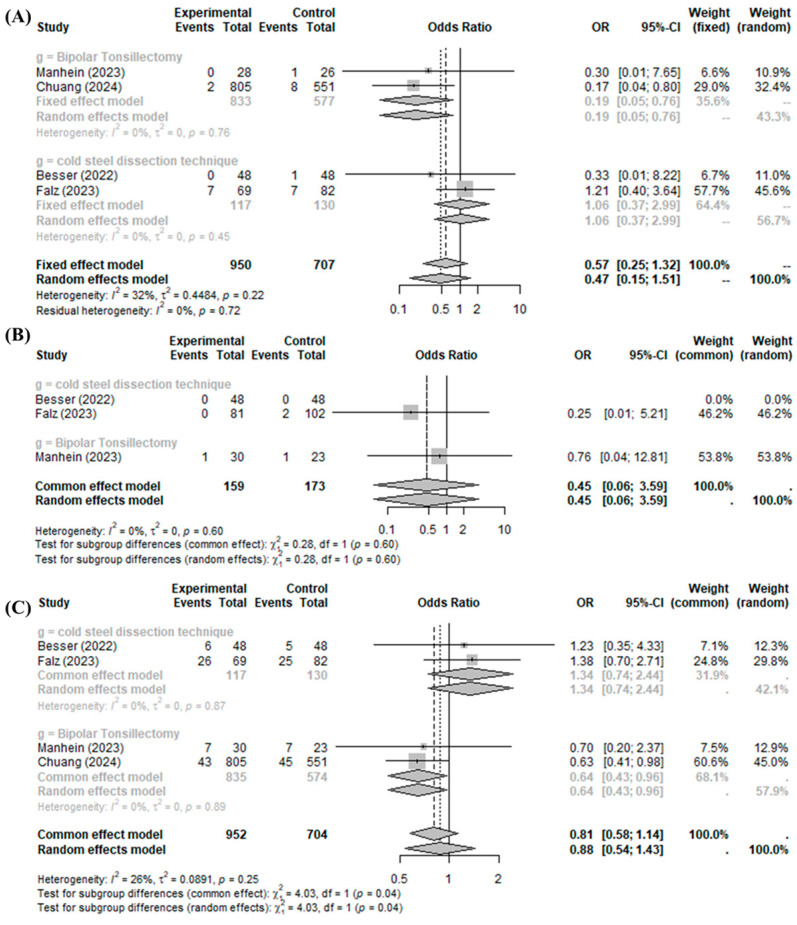
BiZact™ tonsillectomy versus conventional tonsillectomy: odds ratios of (**A**) incidence of postoperative bleeding control in operation room, (**B**) primary bleeding, and (**C**) all postoperative bleeding events. OR: odds ratio, CI: confidence index.

The BiZact™ device did not significantly reduce the incidence of postoperative bleeding control in the operation room (OR 0.5711, 95%CI [0.2476; 1.3173], I^2^ = 32.1%), primary bleeding (OR 0.4514, 95%CI [0.0568; 3.5894], I^2^ = 0.0%), or all postoperative bleeding events (OR 0.8117, 95%CI [0.5796; 1.1368], I^2^ = 26.3%) compared to the control group (Figure 4).

In the subgroup analysis of post-tonsillectomy pain compared to cold steel dissection, the BiZact™ device was not found to significantly reduce the incidence of postoperative bleeding control in the operation room (OR 1.0553, 95%CI [0.3725; 2.9899]), primary bleeding (OR 0.2466, 95%CI [0.0117; 5.2096]), or all postoperative bleeding events (OR 1.3435, 95%CI [0.7400; 2.4391]). Interestingly, the BiZact™ device significantly reduced the incidence of postoperative bleeding control in the operation room (OR 0.1879, 95%CI [0.0463; 0.7629]) and all postoperative bleeding events (OR 0.6410, 95%CI [0.4262; 0.9641]), aside from primary bleeding (OR 0.7586, 95%CI [0.0449; 12.8123]), when compared to bipolar tonsillectomy.

### 3.4. Sensitivity Analysis

A sensitivity analysis was performed by excluding individual studies from the meta-analysis one by one. No one study was found to significantly impact the overall trend.

## 4. Discussion

In this study, we compared the effectiveness of the new BiZact™ tonsillectomy device with cold steel dissection or bipolar tonsillectomy for the first time. Tonsillectomy using the BiZact™ device was shown to involve a significantly reduced intraoperative time compared to the control group. However, its effectiveness in reducing intraoperative bleeding compared with the control group was not significant. Post-tonsillectomy pain did not significantly decrease compared to the control group on day 1, day 3, or later than day 7. Lastly, compared to the control group, BiZact™ involved significantly less post-tonsillectomy bleeding.

We confirmed that the intraoperative time for tonsillectomy using BiZact™ was significantly shorter than that for the control group, and previous studies also confirmed that the surgery time was shorter than those of cold steel dissection, bipolar radiofrequency, or other tonsillectomy devices [15,16,24]. Tonsillectomy using bipolar cauterization takes an average of 15 to 20 min, but that using the BiZact™ device is reported to take an average of 4 to 7.5 min [12,14,15,21,24,25,26].

Our results report that the BiZact™ device did not significantly reduce bleeding risk during or after tonsillectomy. BiZact™ can reduce tissue damage and bleeding by sealing tissue, but the bleeding risk may vary depending on the measurement period. Previous studies have reported that hot dissection techniques such as coblation and bipolar diathermy scissor cause less intraoperative hemorrhage compared to cold steel dissection [18,27,28,29]. BiZact™ has also been reported to involve less intraoperative blood loss [15]. However, it has been reported that coblation and bipolar diathermy scissors have higher postoperative hemorrhage risks compared to cold steel dissection [10,30,31]. Hot dissection was also evaluated as having an increased risk of post-tonsillectomy bleeding compared to cold steel dissection [10,32,33,34]. The post-tonsillectomy bleeding risk of BiZact™ was reported to be around 0.16%−8.6% [5,12,16,35]. Meanwhile, overall post-tonsillectomy bleeding has been reported to be about 4.5% [11]. However, the bleeding risk may vary depending on the age and sex of the patient, as well as the indication for tonsillectomy, such as simple hypertrophied tonsil or recurrent tonsillitis [12,36]. Moreover, there is still no standardized measurement method for blood loss during tonsillectomy and no precise definition of post-tonsillectomy bleeding, so the accuracy of any such comparisons may be low [37].

In this study, postoperative pain after tonsillectomy using the BiZact™ device was not significantly reduced compared to the control group. BiZact™ can reduce tissue damage caused by heat by automatically controlling the energy delivered to tissues and sealing denatured proteins [12,38]. On the other hand, bipolar cauterization seals blood vessels and creates a thrombus, and Ligasure™ generates heat in short, delayed bursts to stop bleeding. Pain may occur due to inadvertent damage to soft tissue from thermal spread [5]. Therefore, it would be ideal to have a device for tonsillectomy that shows high hemostasis with minimal thermal damage [39]. However, it is not yet clear whether such tissue damage caused by heat is directly related to post-tonsillectomy pain [40]. In previous studies, bipolar cauterization or the radiofrequency technique were shown to cause reduced or similar pain compared with cold steel dissection [40]. Tonsillectomy using coblation has a tendency to cause less pain, but there may be differences in the pain assessment methods [27]. The age and gender of the target patients and the surgeon’s skill level may have an impact on post-tonsillectomy pain [41,42,43]. A previous study reported that the return rates of patients who were operated on using the BiZact™ device were lower than those who were operated on through coblation, and this should also be considered [16].

Because the BiZact™ device uses a disposable handpiece, the cost of the technique using this device is higher than that of cold steel dissection. While the bipolar technique costs about USD 30, the BiZact™ technique costs about USD 275, which is about nine times that of the bipolar technique [17]. However, the high cost can be offset by the significantly shorter intraoperative time of BiZact™, which is approximately nine times shorter. BiZact™, which reduces operating room costs, can also be expected to have a long-term cost-saving effect [5]. However, comprehensive cost-effectiveness must be evaluated by comparing the long-term outcome and other complications of tonsillectomy.

This study also had some limitations that should be noted. First, several of the included studies were retrospective studies. There may therefore have been missing information on post-tonsillectomy pain and bleeding in the chart reviews of retrospective studies. Further prospective studies evaluating differences in bleeding risk and post-tonsillectomy pain should thus be included. Second, post-tonsillectomy pain was assessed by different clinics and researchers in each study, and it may have been influenced by the analgesics used during and after tonsillectomy. Third, bleeding risk may vary from study to study. There is a need for a prospective study using the same method for measuring intraoperative blood loss and the exact definition of post-tonsillectomy bleeding. Fourth, the surgical methods used may vary depending on the study. There may be efforts to reduce pain by reducing thermal injury through saline irrigation during tonsillectomy, or attempts to reduce bleeding through local injection. Alternatively, bipolar cauterization may have been used to reduce bleeding. In addition, Besser et al. performed BiZact™ tonsillectomy on one tonsil and cold-steel tonsillectomy on the other tonsil (control group) of the same patients and evaluated the outcomes differently, but the control group may have been different from other studies. Fifth, cold-steel dissection tonsillectomy is not clinically completely classified from bipolar tonsillectomy because it partially uses localized bipolar cauterization. Sixth, it cannot be ruled out that patients used other oral or topical medications for pain control before or after treatment that were not recorded in the medical charts. Lastly, bias related to the funding used in the few studies using BiZact™ is another potential problem. There is a need for additional research without industry sponsorship or any conflicts of interest.

## 5. Conclusions

The BiZact™ device for tonsillectomy significantly reduced operating time, but it was not superior in effectively reducing intraoperative bleeding, postoperative pain, or bleeding. It is necessary to compare the long-term results and additional surgical complications to comprehensively assess cost-effectiveness.

## Figures and Tables

**Table 1:** Summary of included studies.

Study	Study Design	Nation	Total Number of Patients (*n*)	Sex (Female/Male)	Age of Patients (Years, Mean ± Standardized Deviation or Range)	Comparison	Outcomes
Besser (2022) [20]	A single-center, randomized, self-controlled trial	Austria	48	11/17	24.3 ± 6.7	BiZact™ tonsillectomy on one tonsil side versus cold steel dissection tonsillectomy on another side	Operative time, intraoperative bleeding, and postoperative bleeding incidence
Falz (2023) [12]	Retrospective case–control study	Germany	183	51/132	28.3 ± 8.9	Patient with BiZact™ versus cold steel dissection tonsillectomy	Operative time, intraoperative bleeding, postoperative pain, and postoperative bleeding incidence
Yildirim (2023) [22]	Prospective case–control study	Turkey	128	61/67	8.2 (5–14)	Patient with BiZact™ versus bipolar tonsillectomy	Operative time, intraoperative bleeding, and postoperative pain
Manhein (2023) [1]	Prospective, randomized, partly double-blinded study	Norway	65	28/37	25 ± 4.9	Patient with BiZact™ versus bipolar tonsillectomy (conventional non-disposable diathermic scissor)	Operative time, intraoperative bleeding, postoperative pain, and postoperative bleeding incidence
Saleem (2022) [21]	A comparative study using non-probability purposive sampling	India	100	46/54	6–21	Patient with BiZact™ versus cold steel dissection tonsillectomy	Operative time, intraoperative bleeding, and postoperative pain
Chuang (2024) [5]	Retrospective single-surgeon cohort study	Austria	1356	668/688	13.3 (2–61)	Patient with BiZact™ versus bipolar tonsillectomy	Postoperative bleeding incidence

## Data Availability

The raw data of individual articles used in this meta-analysis are included in the main text or Supplementary data.

## Supplementary Materials

The following supporting information can be downloaded at: https://www.mdpi.com/article/10.3390/medicina60091415/s1, Table S1: Quality of individual randomized controlled trial methodology; Table S2: Quality of individual non-randomized controlled trial methodology.
