# Supplementary material for: Effect of the BiZact™ Low-Temperature Dissecting Device on Intra- and Postoperative Morbidities Related to Tonsillectomy—A Systematic Review and Meta-Analysis
Source: Medicina (Kaunas). 2024 Aug 29;60(9):1415. doi: 10.3390/medicina60091415 (PMC11433995; doi:10.3390/medicina60091415)
Supplement: Supplementary file 1 [file medicina-60-01415-s001.zip › medicina-3171337-supplementary.pdf]

Supplementary Table S1. Quality of individual randomized controlled trial methodology.

Study	Random sequence generation	Allocation concealment	Blinding of participants and personnel	Blinding of outcome assessment	Incomplete outcome data addressed	Free of selective reporting	Risk of Bias of randomized studies
Besser (2022)	Yes	Yes	Unclear	Yes	Yes	Yes	Risk of Bias (Unclear)
Manhein (2023)	Unclear	Unclear	high	Yes	Yes	Yes	Risk of Bias (high)

Supplementary Table 2. Quality of individual non-randomized controlled trial methodology.

Study	Selection[a]				Comparability[b]		Exposure[c]			Newcastle-Ottawa Scale score
	1	2	3	4	5A	5B	6	7	8	
Falz (2023)	Yes	No	Yes	Yes	No	No	Yes	Yes	Yes	6
Yildirim (2023)	Yes	No	No	Yes	Yes	Yes	Yes	Yes	Yes	7
Saleem (2022)	Yes	Yes	Yes	Yes	No	No	Yes	Yes	Yes	7
Chuang (2024)	Yes	Yes	Yes	Yes	No	No	Yes	Yes	Yes	7

A star rating system was used to indicate the quality of a study, with the maximum rating of nine stars. A study could be awarded a maximum of one star for each numbered item within the selection and exposure categories. a: Selection (4 items): adequacy of case definition; representativeness of cases; selection of controls; definition of controls. b: Comparability (1 item): comparability of cases and controls on the basis of design or analysis. c: Exposure (3 items): ascertainment of exposure; same method of ascertainment used for cases and controls; and non-response rate (same rate for both groups).
